# Supplementary material for: Diagnostic Accuracy of DNA-Methylation in Detection of Cervical Dysplasia: Findings from a Population-Based Screening Program
Source: Cancers (Basel). 2024 May 23;16(11):1986. doi: 10.3390/cancers16111986 (PMC11171015; doi:10.3390/cancers16111986)
Supplement: Supplementary file 1 [file cancers-16-01986-s001.zip › cancers-3005105-supplementary.pdf]

Supplementary materials S1

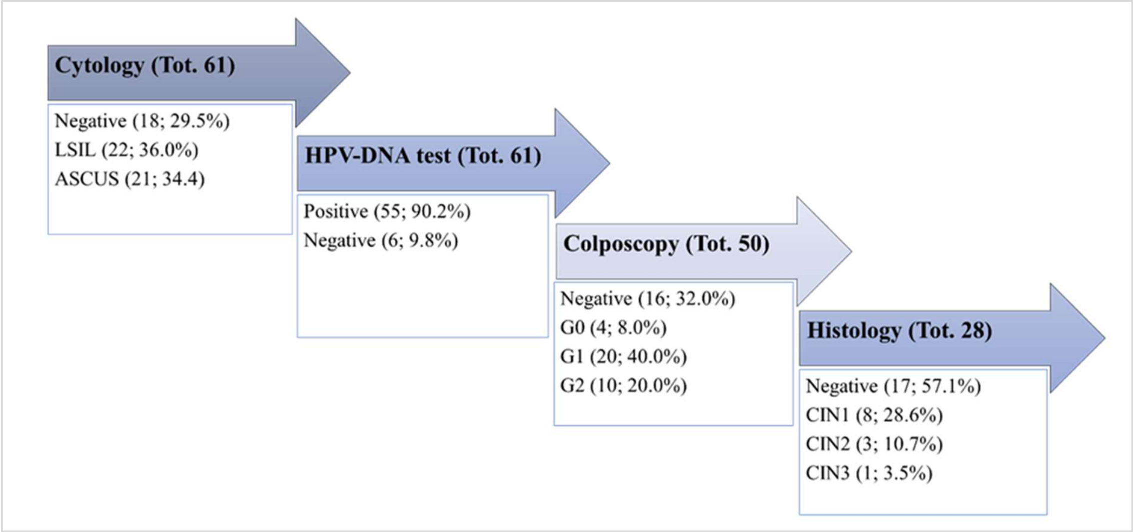

S1_Figure 2. Workflow of cervical screening program and characteristics of sample set.

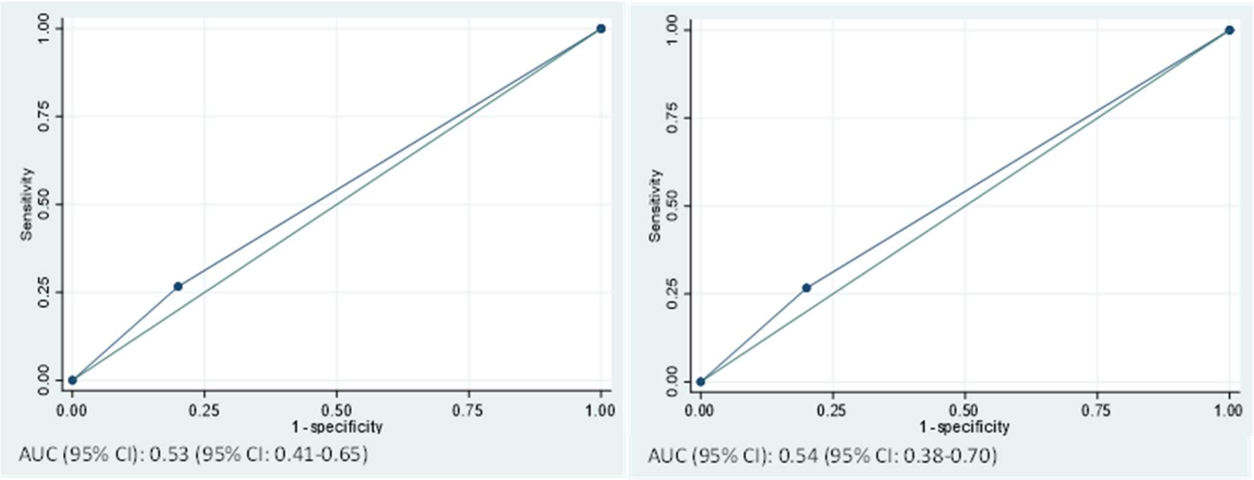

S1_Figure 1a, 1b: Receiver Operating Characteristic (ROC) Curve analysis and associated AUC values of the methylation test alone (a) and combined with HPV-DNA test (b) in identification of positive colposcopy cases (i.e., ≥1 G1).
